# The Convulsive Cough of Puberty

**Published:** 1890-12-20

**Authors:** Andrew Clark


### The Convulsive Cough of Puberty.

By Sir Andrew Clark, M.D., F.R.S.

At the Medical Society on Monday evening Sir Andrew Clark read a paper on the convulsive cough of puberty. Sir Andrew soon made it plain that he considered himself to be dealing with a definite and particular series of phenomena, quite distinct and separate from those characterising any other disease. The convulsive cough of puberty he did not regard as a mere variety of the ordinary nervous cough, but as what we may call, for want of a better word, a patho-physiological entity. He first described four cases; and the clearness and precision with which he presented a picture of each were remarkable, and eminently characteristic. About thirty years ago a boy at the age of puberty was brought to him from Rochester. The boy had a cough which was said to be loud, dry, clanging, and convulsive. At times it would rise into a howl like that of a dog, or it would degenerate into a peculiar sound resembling the barking of the same animal. During the paroxysms the patient was sometimes thrown into a state of the greatest and most distressing excitement; the arms and other parts of the body were convulsed, and the eyes seemed as if they would start from the head. This condition continued for thirteen months. In 1868, a second boy, the son of a clergyman, was brought to Sir Andrew. He had similar symptoms, together with some loss of flesh. So terrible and alarming were his fits of coughing that no school would keep him. Both his parents were nervous and excitable persons. In 1875 a clergyman's daughter, thirteen years old, was seen by Sir Andrew. Her parents too were very nervous. Her paroxysms of coughing so distressed and excited both the girl and those who were near her, that whenever an attack came on everybody but the mother would promptly leave the room. Even the mother herself often felt it all but impossible to remain. This case became so desperate that a surgical operation was proposed, and the instruments were brought and laid out before the patient's eyes. She coughed no more after that. The cure was immediate and final. A fourth case, which was seen two years ago, and which, in addition to the cough, had some quite perceptibl· dry rales in parts of the lungs and bronchi, was also described.

The four cases mentioned constituted the concrete starting point of Sir Andrew's argument. He was not, he said, introducing a new disease to the notice of the society, though he was calling attention to a class of cases very important in themselves, and to which very few references had been made in medical literature. What was the nature of the physiological and pathological changes which set up those alarming and distressing symptoms? Two facts had to be borne in mind: First, that in none of the cases was there any discoverable lesion, except perhaps of the most trifling kind; and, secondly, that the patient almost or quite invariably recovered, whatever treatment might be employed. How, then, were the phenomena to be explained? Sir Andrew thought that the profound changes incident to sexual evolution and development were sufficient explanation. At the age of puberty, the boy becomes a man; the girl a woman. The degree of development which marks this transition is great and universal. The whole physiological system is strikingly influenced by it; and especially the nervous portion of the organism. In the case of certain individuals of abnormally nervous temperament and inheritance, the disturbance is so great as to produce a condition of unstable equilibrium. Muscular control is impaired, and impaired unequally. The discharges of nervous energy are no longer in strict order and succession. What part of the nervous system is at fault, the central or the peripheral? asked Sir Andrew. Both undoubtedly; and the central probably more than the peripheral. But the peripheral was not to be ignored, especially after the coughing habit had been established. The disease, said Sir Andrew, had its origin in conditions that were both physical and psychical, mental and moral. He wished therefore to present to his audience not a succession of symptoms alone, but persons, histories, and stages in physical, mental, and moral evolution.

What is to be done with such cases? What treatment is to be adopted? The patient is to be treated, and not merely the cough. The treatment must be specially hygienic, but medicinal remedies of a locally soothing and nerve-steadying kind were of some service. Mental and moral regimen was of prime importance. The patient should be taught to exercise the greatest possible control over himself. That such control could in certain cases be so complete as to terminate the affection was proved by the case of the clergyman's daughter, who was effectually cured by the promise of a surgical operation, and the sight of the laid-out instruments. Such drugs as morphia or cocaine, with glycerine of borax, applied locally, and bromide of quinine and iron, or iron with belladonna and valerianate of zinc, given internally, he had found decidedly useful as adjuncts. They had not cut short the disease, but they had helped the patient through it. Of more importance, however, than drugs Sir Andrew seemed to consider the diet and regimen. A simple diet, a very simple diet, he insisted upon; and no alcohol. Some physicians, he said, were very fond of recommending sea voyages. That was not his judgment on the matter; the curative results of the voyage were seldom satisfactory, whilst certain other results were decidedly injurious. Boys at the pubertal age often learnt habits at sea which, once acquired, were extremely difficult to eradicate, and which produced both moral and physical deteriorations of a very unhappy kind. Keep your boys at home, was Sir Andrew's advice, and trust to moral and physical regimen, to simple diet, and tonic and soothing drugs, and to time. The physician can seldom shorten the disease in any appreciable degree; but he can always with judicious management conduct the case to a happy termination.

The discussion which followed was interesting, and sometimes, even in that grave assembly, a little amusing. Dr. Althaus, for example, first paid Sir Andrew Clark a profusion of compliments, and then questioned whether he had applied the results of the atest physiological research to the explanation of the phenomena of convulsive cough. Sir Andrew bided his time, and then turned the tables upon the physiological German by quoting still more recent researches, and regretfully admitting that their illuminating power had not been such as to make all difficulties vanish. Another gentleman, whose name did not transpire, said he had listened with much interest both to the reading of the paper and to the discussion, but he was sorry to say that he did not find himself any wiser than before. That was, to say the least, ungrateful, and, perhaps, showed a want of understanding on the part of the speaker. For Sir Andrew Clark in his paper certainly dealt with facts; with facts not common, and yet such as may be required to be dealt with at any moment in every consulting room; with facts, too, of undeniable importance both to the patient, his friends, and the medical attendant; and he dealt with those facts in a lucid, judicial, and practical way which left nothing to be desired. Any hearer, therefore, who learnt nothing had himself to thank. Other speakers included Dr. Stephen Mackenzie, Dr. Gowers, Dr. de Haviland Hall, Dr. Percy Kidd, Dr. Norman Kerr, and Dr. Thomas Maclagan. Most of those speakers concurred with Sir Andrew Clark both as to the etiology of the disease under discussion and as to its treatment. The meeting was of an unusually interesting and practical character.

